# Influence of social exclusion on aggressive behavior among specialized school students: the mediating roles of relative deprivation and deviant peer affiliation

**DOI:** 10.3389/fpsyg.2026.1855838

**Published:** 2026-09-21

**Authors:** Miaomiao Tao, Xue Zhang, Linran Zhang, Xianglan Zhang, Jingxuan Shi, Zexiang You

**Affiliations:** 1 College of Education, Ludong University, Yantai, Shandong, China; 2 Institute for Education and Treatment of Problematic Youth, Ludong University, Yantai, Shandong, China; 3 Center for Juvenile Delinquency Prevention and Correction, The National Police University for Criminal Justice, Baoding, Hebei, China

**Keywords:** aggressive behavior, deviant peer affiliation, relative deprivation, social exclusion, specialized school students

## Abstract

**Objective:**

This study examined the association between social exclusion and aggressive behavior among students in specialized schools and investigated the mediating roles of relative deprivation and deviant peer affiliation.

**Method:**

A total of 491 students from specialized schools completed the Ostracism Experience Scale for Adolescents, Relative Deprivation Questionnaire, Deviant Peer Affiliation Questionnaire, and Buss–Perry Aggression Questionnaire.

**Results:**

Social exclusion was significantly and positively associated with aggressive behavior among students in specialized schools. Relative deprivation and deviant peer affiliation independently mediated the association between social exclusion and aggressive behavior. In addition, relative deprivation and deviant peer affiliation formed a significant serial mediating pathway between social exclusion and aggressive behavior.

**Conclusion:**

Social exclusion was directly associated with aggressive behavior and was also indirectly associated with aggressive behavior through relative deprivation and deviant peer affiliation. These findings provide practical implications for the prevention and intervention of aggressive behavior among students in specialized schools.

## 1 Introduction

In recent years, youth violence has become a serious social concern worldwide. The World Health Organization has identified violence as an important cause of death and injury among young people. Worldwide, approximately 200,000 homicides occur each year among youth and young adults aged 10–29 years (World Health Organization, 2015). Aggressive behavior is an important precursor to and risk factor for youth violence. Previous studies have shown that aggression is associated with mental health problems among adolescents, including depression and anxiety (Li et al., 2025; Yang et al., 2024). Higher levels of aggression during childhood and adolescence may also increase the risk of subsequent delinquent and criminal behavior (Huesmann et al., 2002). In China, specialized schools evolved from reform schools and are primarily responsible for providing specialized education and correction to minors who have engaged in serious misconduct. Specialized education is an important protective measure designed to educate and correct minors with serious misconduct, and it differs in nature from criminal punishment. According to the Law of the People’s Republic of China on the Prevention of Juvenile Delinquency, serious misconduct includes acts prescribed by criminal law for which minors are not subject to criminal punishment because they have not reached the age of criminal responsibility, as well as acts that seriously endanger society, such as group fighting, intentional injury, and theft. Students in specialized schools are minors who, following an assessment and formal decision, are placed in these schools to receive specialized education because of such serious misconduct. Comparative research has shown that students in specialized schools exhibit higher levels of aggression than students in regular schools (Li et al., 2017). Among students in specialized schools, aggression may undermine the effectiveness of education and correction and increase the risk that problem behaviors will persist or escalate. Therefore, examining the factors associated with aggression in this population is important for improving education and correction, enhancing the quality of specialized education, and preventing youth violence and delinquency. Adolescence is a critical period marked by rapid development in cognition, self-regulation, and social relationships. When facing negative interpersonal situations such as rejection, some adolescents may respond with aggressive behavior (Quarmley et al., 2023). Aggressive behavior refers to any behavior intended to cause harm or pain to others (Anderson and Bushman, 2002). The General Aggression Model (GAM) provides a systematic theoretical framework for understanding how negative situations are associated with aggressive behavior. According to the GAM, individual and situational factors serve as input variables that influence internal states, including cognition, affect, and physiological arousal. These internal states further shape appraisal and decision-making processes, which may ultimately result in aggressive or nonaggressive behavior (Allen et al., 2018). Social exclusion is a typical negative interpersonal experience and can be viewed as an adverse situational input within the GAM. It refers to experiences of being ignored, rejected, or excluded by others or groups, which may threaten individuals’ needs for belonging and social connection (Rajchert and Winiewski, 2016; Williams, 2009). A meta-analysis of 92 studies with 99 independent samples found a moderate positive association between social exclusion and aggressive behavior, indicating that individuals who experience greater social exclusion tend to show higher levels of aggression (Jin et al., 2023). In addition, experiences of social exclusion may activate hostile thoughts and hostile attribution, making individuals more likely to interpret ambiguous social information as hostile and thereby increasing aggressive tendencies. Negative emotions such as anger elicited by social exclusion may also further promote aggressive responses (DeWall et al., 2009; Shao and Zhang, 2021).

Social exclusion may also increase the likelihood of aggressive responses by altering individuals’ internal states. Relative deprivation refers to a subjective cognitive and emotional experience in which individuals perceive themselves to be disadvantaged through comparisons with relevant individuals or groups, which may further give rise to negative emotions such as anger and perceived unfairness (Pettigrew, 2016; Smith et al., 2012). Previous studies have found a significant positive association between social exclusion and relative deprivation (Duran Mucuk and Şahin, 2025). When individuals perceive themselves as being excluded from positive peer interactions or group belonging, they may become more aware of their relatively disadvantaged position through social comparison, thereby developing a sense of deprivation. Thus, social exclusion may serve as an important antecedent of relative deprivation. Relative deprivation may, in turn, be associated with aggressive behavior. Previous research has shown that individuals who perceive higher levels of deprivation are more likely to engage in aggressive behavior (Ding et al., 2020; Wang et al., 2023; Tong and Jiang, 2026). Within the framework of the General Aggression Model, relative deprivation can be viewed as an important internal state that may arise when individuals experience social exclusion as an adverse situation. As a negative emotional experience, relative deprivation is often accompanied by strong feelings of unfairness, anger, and resentment. When these feelings of deprivation accumulate, individuals may be more likely to use aggressive behavior as a means of compensation or emotional release. Therefore, relative deprivation may mediate the association between social exclusion and aggressive behavior.

In addition to influencing individuals’ internal psychological states, social exclusion may also indirectly increase the likelihood of aggressive behavior by changing adolescents’ patterns of peer affiliation. For adolescents, peer groups are an important source of social acceptance, belonging, and identity. Deviant peer affiliation refers to associations with peers who violate social norms and engage in similar activities and behaviors (Dishion and Tipsord, 2011). When adolescents repeatedly experience social exclusion, their needs for belonging and acceptance may remain unmet. This may encourage them to seek alternative peer relationships that provide recognition, acceptance, and a sense of belonging. Previous studies have shown that exclusion-related experiences, such as peer rejection and bullying victimization, are closely associated with deviant peer affiliation (Lansford et al., 2014; Rudolph et al., 2014; Chen et al., 2015; Dong et al., 2023). Social learning theory suggests that individuals can acquire and maintain behavioral patterns through observation, imitation, and social reinforcement (Akers, 2009; Dishion and Tipsord, 2011). Adolescents affiliated with deviant peers may be repeatedly exposed to aggressive behavior and related behavioral norms. Through observation and imitation, they may acquire similar behavioral patterns, which may be further reinforced by peer approval and support. This process may increase the likelihood of aggressive behavior (Lin et al., 2022; Li et al., 2024).

Notably, relative deprivation and deviant peer affiliation may not operate independently but may instead form a sequential process. Higher levels of relative deprivation indicate that individuals are more likely to perceive themselves as disadvantaged and to experience unfairness and negative emotions. These experiences may further increase their tendency to seek alternative sources of social support and group belonging. For some adolescents, deviant peer groups may be perceived as offering greater social status and autonomy (Tian et al., 2025). Such groups may also provide a certain degree of acceptance, recognition, and belonging, thereby serving as an important source of social connection (Williams, 2007). Therefore, adolescents with higher levels of relative deprivation may be more likely to affiliate with deviant peers to obtain the social status, support, and sense of belonging that are not fully satisfied in their existing relationships (Callan et al., 2008; Yang et al., 2021). A serial mediation analysis conducted among students in specialized schools found that relative deprivation was significantly and positively associated with deviant peer affiliation (Wang et al., 2026). Taken together, relative deprivation may further increase the likelihood of adolescents affiliating with deviant peers, and the two factors may form a serial mediating pathway between social exclusion and aggressive behavior.

As noted above, this study focuses on students in specialized schools and, based on the General Aggression Model and Social Learning Theory, examines the association between social exclusion and aggressive behavior. It further examines the mediating and serial mediating roles of relative deprivation and deviant peer affiliation in this association. Accordingly, the following hypotheses are proposed:

*H1*: Social exclusion is positively associated with aggressive behavior among specialized school students.

*H2*: Relative deprivation mediates the association between social exclusion and aggressive behavior.

*H3*: Deviant peer affiliation mediates the association between social exclusion and aggressive behavior.

*H4*: Relative deprivation and deviant peer affiliation sequentially mediate the association between social exclusion and aggressive behavior.

## 2 Method

### 2.1 Participants and sampling procedure

In this study, students in specialized schools were defined as minors who, following an assessment and a formal decision, had been placed in these schools to receive specialized education because of serious misconduct. Given the characteristics of the study population and the limited number of specialized schools, purposive sampling was employed. Taking into account geographical distribution, schools’ willingness to participate, and the feasibility of data collection, three specialized schools located in Hunan, Shandong, and Hainan provinces, China, were selected. After the schools had been selected, all students who met the inclusion criteria were invited to participate. To facilitate centralized data collection and minimize disruption to normal teaching activities, the questionnaire survey was administered by class in accordance with the arrangements of each school. Based on the minimum sample size criterion of at least five participants per questionnaire item (Tinsley and Tinsley, 1987), the 43-item questionnaire used in this study required at least 215 valid participants. Allowing for an estimated 20% rate of invalid or incomplete responses, the target sample size was approximately 269 participants.

The inclusion criteria were as follows: (a) enrollment in one of the specialized schools participating in the study; (b) ability to understand the questionnaire and complete it independently; and (c) voluntary agreement to participate in the study. Questionnaires with substantial missing data, obvious patterned responses, or invariant responses were excluded. A total of 520 questionnaires were distributed, and 491 valid questionnaires were obtained, yielding a valid response rate of 94.42%. The final sample included 470 male students (95.72%) and 21 female students (4.28%). Participants ranged in age from 10 to 17 years (*M* = 14.88, SD = 1.19). In addition, 53.56% of the students had a history of violence-related problem behaviors, such as group fighting, provoking disturbances, and robbery. A total of 60.49% had a history of property-related problem behaviors, such as theft and fraud, and 20.78% had a history of other types of problem behaviors. These categories were not mutually exclusive; therefore, the same student could be included in more than one category.

Prior to data collection, approval was obtained from the relevant administrative authorities of the participating schools, and informed consent was obtained from the participants’ parents or legal guardians. The study was conducted in accordance with the principles of the Declaration of Helsinki.

The study was approved by the Institutional Review Board of Ludong University (Approval No. LDU-IRB2026041003).

### 2.2 Measures

#### 2.2.1 The Ostracism Experience Scale for Adolescents (OES-A)

The OES-A was developed by Gilman et al. (2013) and subsequently adapted by Zhang et al. (2018). In the adaptation study, the OES-A demonstrated good structural validity among Chinese adolescents (*χ*^2^/d*f* = 2.09, *p* < 0.001, CFI = 0.98, TLI = 0.98, and RMSEA = 0.04) and acceptable reliability (Cronbach’s *α* = 0.76; six-month test–retest reliability coefficient = 0.65). It consists of 11 items across two dimensions: neglect and rejection. Example items include “Other people often come to my home to play with me” and “When everyone is talking together, other people often ignore me.” Items are rated on a five-point Likert scale ranging from 1 (never) to 5 (always). Items in the rejection dimension are reverse-scored, such that higher total scores indicate higher levels of social exclusion. In the present study, the complete 11-item version was used, with no items omitted or modified. The scale demonstrated acceptable internal consistency in the current sample (Cronbach’s *α* = 0.76).

#### 2.2.2 Relative Deprivation Questionnaire (RDQ)

The present study used the RDQ developed by Ma (2012). It assesses individuals’ perceived disparity when comparing themselves with a reference group. It consists of four items. An example item is “Compared with the effort and work I have put in, my life should be better than it is now.” Items are rated on a 6-point Likert scale, with higher scores indicating higher levels of relative deprivation. The questionnaire has been used in studies involving Chinese adolescents, and previous research has supported its good structural validity (Wu and Zhang, 2024) and internal consistency (Kong et al., 2024; Yang et al., 2021). In the present study, the complete four-item version was used, with no items omitted or modified. The questionnaire demonstrated acceptable internal consistency in the current study (Cronbach’s *α* = 0.71).

#### 2.2.3 Deviant Peer Affiliation Questionnaire (DPAQ)

The DPAQ adapted by Li et al. (2013) was used to assess deviant peer affiliation among students in specialized schools. It consists of eight items assessing deviant peer behaviors, such as truancy and drinking. Items are rated on a five-point Likert scale, with higher scores indicating higher levels of deviant peer affiliation. The questionnaire has demonstrated good structural validity (Wang et al., 2020) and internal consistency (Li et al., 2023; Liu et al., 2025; Wang et al., 2026) among Chinese adolescents. No items were deleted or modified in the present study. In the current sample, the questionnaire demonstrated good internal consistency (Cronbach’s *α* = 0.80).

#### 2.2.4 Buss–Perry Aggression Questionnaire (BPAQ)

Aggressive behavior in adolescents was assessed using the revised BPAQ (Liu et al., 2009). The revision study reported good structural validity (KMO = 0.898, *χ*^2^ = 2886.45, d*f* = 231, *p* < 0.01) and reliability (Cronbach’s *α* = 0.84, test–retest reliability = 0.92) among Chinese adolescents. It consists of 20 items across four dimensions: physical aggression, hostility, anger, and substitute aggression. Among them, physical aggression reflects aggressive behavioral manifestations, anger belongs to emotional expression, and hostility belongs to cognitive expression. Alternative aggression is manifested externally as causing harm to objects or indirectly attacking people or objects with words, and internally as suppressed negative emotions. An example item is “Some of my friends think I have a hot temper.” The scale uses a 5-point Likert format, with higher scores indicating greater levels of aggression. No items were deleted or modified in the present study. The scale demonstrated excellent internal consistency in the present study (Cronbach’s *α* = 0.91).

### 2.3 Data analysis

Data analyses were conducted using SPSS 31.0 and Mplus 8.3. First, descriptive statistics, correlation analyses, and Harman’s single-factor test were performed in SPSS 31.0. Harman’s single-factor test was used to assess potential common method bias (Zhou and Long, 2004). The results showed that 10 factors had eigenvalues greater than 1, and the first factor accounted for 20.00% of the total variance, which was below the recommended threshold of 40%. These results suggest that common method bias was unlikely to pose a serious threat to the validity of the findings. Second, a serial mediation model was tested in Mplus 8.3 to examine the mediating roles of relative deprivation and deviant peer affiliation in the association between social exclusion and aggressive behavior.

## 3 Results

### 3.1 Correlation analyses

The observed score ranges, means, standard deviations, and correlations for all study variables are presented in Table 1. The absolute skewness values ranged from 0.01 to 0.50, and the absolute kurtosis values ranged from 0.11 to 1.02. These values indicated no substantial departures from normality. Because the scale scores were calculated as mean item scores, could be treated as continuous variables, and were approximately normally distributed, Pearson correlation analysis was used.

**Table 1:** Descriptive statistics and correlations (*N* = 491).

Variables	Range	*M*	SD	1	2	3	4
1. Social exclusion	1.09–5.00	2.46	0.58	1			
2. Relative deprivation	1.00–6.00	3.40	0.96	0.13^**^	1		
3. Deviant peer affiliation	1.25–5.00	3.13	0.80	0.18^***^	0.23^***^	1	
4. Aggressive behavior	1.00–5.00	2.93	0.75	0.19^***^	0.24^***^	0.42^***^	1

Range represents the observed minimum and maximum mean scores. All scale scores were calculated as mean item scores. Pearson correlation coefficients are reported. *p < 0.05; **p < 0.01; ***p < 0.001.

Descriptive statistics and correlations among the study variables are presented in Table 1. According to Cohen’s (1988) guidelines, social exclusion showed small positive correlations with relative deprivation (*r* = 0.13, *p* < 0.01), deviant peer affiliation (*r* = 0.18, *p* < 0.001), and aggressive behavior (*r* = 0.19, *p* < 0.001). Relative deprivation also showed small positive correlations with deviant peer affiliation (*r* = 0.23, *p* < 0.001) and aggressive behavior (*r* = 0.24, *p* < 0.001). In contrast, deviant peer affiliation was moderately and positively correlated with aggressive behavior (*r* = 0.42, *p* < 0.001).

### 3.2 Chain mediation analysis

The serial mediation model was tested using path analysis. The specified path model was saturated (d*f* = 0); therefore, global model fit indices were not interpreted. The path analysis results are presented in Table 2, and the overall model is shown in Figure 1. The path from social exclusion to relative deprivation was significant and positive (*β* = 0.12, *t* = 2.67, *p* < 0.01). The paths from social exclusion to deviant peer affiliation (*β* = 0.15, *t* = 3.38, *p* < 0.01) and aggressive behavior (*β* = 0.11, *t* = 2.34, *p* < 0.05) were also significant and positive. Relative deprivation showed significant positive paths to deviant peer affiliation (*β* = 0.18, *t* = 4.15, *p* < 0.001) and aggressive behavior (*β* = 0.13, *t* = 2.80, *p* < 0.01). Finally, the path from deviant peer affiliation to aggressive behavior was significant and positive (*β* = 0.36, *t* = 7.96, *p* < 0.001).

**Table 2:** Regression analysis of the serial mediation model between social exclusion and aggressive behavior.

Variables	Relative deprivation				Deviant peer affiliation				Aggressive behavior			
	*β*	*t*	SE	95% CI	*β*	*t*	SE	95% CI	*β*	*t*	SE	95% CI
Gender	0.10	2.32	0.04	[0.014, 0.180]	0.07	1.38	0.05	[−0.031, 0.162]	0.11	2.03	0.06	[0.002, 0.214]
Age	0.10	2.26	0.04	[0.013, 0.183]	0.20	4.72	0.04	[0.113, 0.274]	0.02	0.57	0.04	[−0.059, 0.109]
Social exclusion	0.12	2.67	0.05	[0.035, 0.219]	0.15	3.38	0.04	[0.060, 0.234]	0.11	2.34	0.05	[0.015, 0.201]
Relative deprivation					0.18	4.15	0.04	[0.096, 0.269]	0.13	2.80	0.05	[0.042, 0.230]
Deviant peer affiliation									0.36	7.96	0.05	[0.269, 0.443]
*R* ^2^	0.03				0.12				0.22			

All coefficients are standardized. ***p < 0.001, **p < 0.01, *p < 0.05.

**Figure 1:**
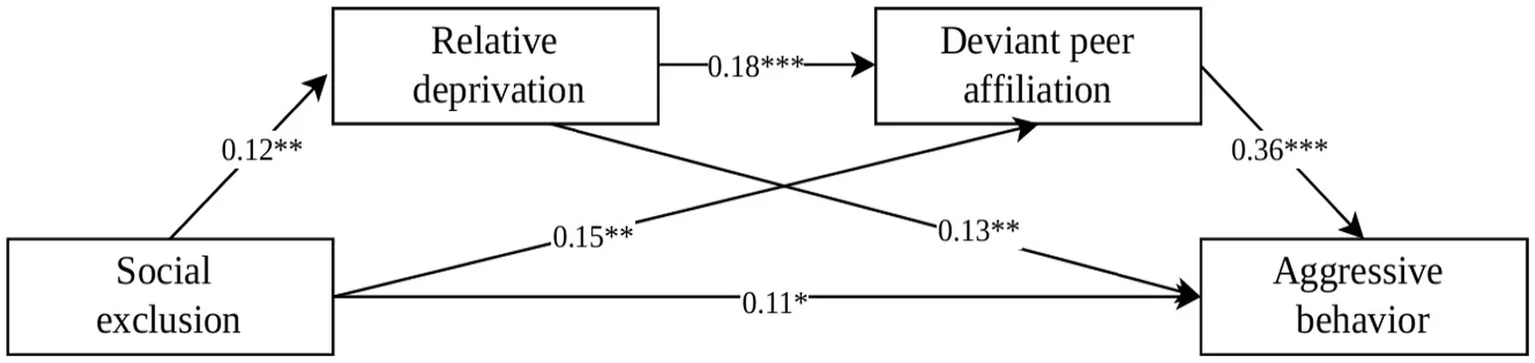
The chain mediation model and *β* of each path. ***p < 0.001; **p < 0.01; *p < 0.05.

To further examine the mediating roles of relative deprivation and deviant peer affiliation in the association between social exclusion and aggressive behavior, a bias-corrected bootstrap procedure with 5,000 resamples was conducted in Mplus. An indirect effect was considered statistically significant when its 95% confidence interval did not include zero. All effects reported in Table 3 are STDYX-standardized effect estimates from Mplus rather than unstandardized coefficients. The indirect effect through relative deprivation was 0.016, accounting for 8.47% of the total effect. The indirect effect through deviant peer affiliation was 0.054, accounting for 28.57% of the total effect. The serial indirect effect through relative deprivation and deviant peer affiliation was 0.008, accounting for 4.23% of the total effect. The 95% confidence intervals for all three indirect effects did not include zero, indicating that both specific indirect effects and the serial indirect effect were statistically significant. The indirect effect through deviant peer affiliation was the largest, followed by the indirect effect through relative deprivation, whereas the serial indirect effect was the smallest.

**Table 3:** Bootstrapping output for serial mediation model.

Pathway	Standardized effect-size	SE	95% CI	Relative effect
Social exclusion → Relative deprivation → Aggressive behavior	0.016	0.010	[0.003, 0.042]	8.47%
Social exclusion → Deviant peer affiliation → Aggressive behavior	0.054	0.017	[0.022, 0.090]	28.57%
Social exclusion → Relative deprivation → Deviant peer affiliation → Aggressive behavior	0.008	0.003	[0.003, 0.017]	4.23%
Total indirect effect	0.078	0.021	[0.041, 0.122]	41.27%

## 4 Discussion

### 4.1 The relationship between social exclusion and aggressive behavior

The present study found that social exclusion was significantly and positively associated with aggressive behavior among specialized school students, thereby supporting Hypothesis 1. This finding is consistent with previous studies showing a positive association between social exclusion and aggressive behavior (Qiu et al., 2025; Yu et al., 2025; Zhao et al., 2025). One possible explanation is that social exclusion, as a typical negative life event, disrupts individuals’ emotional and cognitive processing. It may influence cognition, affect, and physiological arousal, thereby increasing the likelihood of aggressive responses (Allen et al., 2018). In addition, social exclusion can disrupt social connectedness and elicit “social pain” experiences that resemble physical pain, which may in turn activate hostile cognitions and aggressive tendencies among adolescents and elevate the risk of aggression (Bai and Yang, 2025; Mo et al., 2024). For students in specialized schools, who often have a history of problem behaviors and frequently come from left-behind, single-parent, or conflictual family environments, these experiences make them more likely to be ignored or marginalized in daily interactions, thereby intensifying experiences of social exclusion and further increasing the probability of aggressive behavior (Zhang and Li, 2025). Consequently, they are more likely to resort to aggressive behavior as a response to social exclusion.

### 4.2 The mediating role of relative deprivation

Second, the present study found that relative deprivation mediated the association between social exclusion and aggressive behavior, supporting Hypothesis 2. This finding is consistent with previous studies (Yu et al., 2025). It suggests that social exclusion is associated with aggressive behavior not only directly but also indirectly through relative deprivation. Experiences of social exclusion among students in specialized schools may heighten their awareness of differences between themselves and others in social acceptance and group status. When these differences are perceived as signs of disadvantage or unfair treatment, stronger feelings of relative deprivation may arise. This finding is also consistent with the General Aggression Model. The negative cognitions and emotions involved in relative deprivation may further influence individuals’ appraisal of the current situation and the consequences of their behavior, thereby increasing the likelihood of aggressive behavior (Anderson and Bushman, 2002; Shao and Zhang, 2021). Therefore, when seeking to understand aggressive behavior among students in specialized schools, attention should be paid not only to their experiences of social exclusion but also to the relative deprivation that may arise from these experiences.

### 4.3 The mediating role of deviant peer affiliation

In addition, the present study found that deviant peer affiliation mediated the association between social exclusion and aggressive behavior. Social exclusion involves various interpersonal threats and negative experiences. When adolescents repeatedly experience social exclusion, their needs for belonging and acceptance may not be fully satisfied. Under such circumstances, they may be more likely to seek alternative peer groups that can provide acceptance, recognition, and emotional support. Interactions with deviant peers often involve group-based activities, and repeated contact and shared activities may strengthen emotional bonds among group members. To some extent, these relationships may compensate for the unmet need for belonging resulting from social exclusion (Liu and Su, 2023; Yang and Huang, 2022). Therefore, individuals who experience higher levels of social exclusion may be more likely to affiliate with deviant peers. Previous studies have also shown that deviant peer affiliation is closely associated with aggressive behavior (Cairns et al., 1988; Lin et al., 2020; Gao et al., 2015). Through frequent interactions with deviant peers, adolescents may observe and imitate peers’ aggressive behavior and gradually acquire similar behavioral patterns. In addition, behavioral displays, peer pressure, and social reinforcement within peer interactions may further strengthen adolescents’ acceptance of deviant behavior and increase the likelihood of engaging in aggression (Dishion and Tipsord, 2011).

Notably, among the two indirect pathways through relative deprivation and deviant peer affiliation, the indirect effect through deviant peer affiliation was larger. This difference in effect size may reflect the different processes represented by the two mediators. Relative deprivation mainly reflects the internal cognitive and emotional processes that follow experiences of social exclusion (Smith et al., 2012), whereas deviant peer affiliation involves more sustained peer interaction and social reinforcement processes (Dishion and Tipsord, 2011). Therefore, when understanding the association between social exclusion and aggressive behavior among students in specialized schools, deviant peer affiliation may represent a particularly important social factor.

### 4.4 Serial mediation of relative deprivation and deviant peer affiliation

The present study also identified a serial mediating pathway through relative deprivation and deviant peer affiliation between social exclusion and aggressive behavior, supporting Hypothesis 4. This finding suggests that the relative deprivation associated with social exclusion may not remain only at the level of adolescents’ internal experiences but may also extend to their peer relationships. When adolescents experience higher levels of relative deprivation, they may perceive greater psychological stress, lower social status, and stronger feelings of unfairness (Tian et al., 2025). As a result, they may seek affiliation with deviant peers as a way to relieve psychological tension. Adolescents with higher levels of relative deprivation may therefore be more likely to affiliate with deviant peers who are subject to fewer behavioral constraints and are perceived as having greater autonomy and social status (Callan et al., 2008; Yang et al., 2021). Overall, these findings suggest that the association between social exclusion and aggressive behavior may involve a process that extends from internal psychological experiences to external peer relationships, providing a more integrated explanation of aggressive behavior among students in specialized schools.

It is important to note that although the serial indirect effect through relative deprivation and deviant peer affiliation was statistically significant, its standardized estimate was only 0.008. It accounted for 4.23% of the total effect and was substantially smaller than the other two indirect effects. This difference may reflect variation in how students respond to social exclusion. For some students, relative deprivation may be more directly associated with aggressive behavior. For others, a thwarted need to belong may be directly related to deviant peer affiliation without necessarily being accompanied by a strong sense of relative deprivation. The full serial association may emerge only when relative deprivation associated with social exclusion is further linked to changes in peer affiliation, which are then related to aggressive behavior through sustained interactions with deviant peers. Because this pathway involves several sequential psychological and social processes, each of which may be influenced by individual and contextual factors, its overall effect may be smaller than those of the shorter indirect pathways.

In addition, the present study found that gender and age showed different patterns of associations with the study variables. Gender was significantly associated with relative deprivation and aggressive behavior, but not with deviant peer affiliation. This may be because students in specialized schools share similar school and peer environments, and their peer interactions may be shaped more by common contextual factors. Age was significantly and positively associated with relative deprivation and deviant peer affiliation, possibly because older students have more experience with social comparison and peer interaction. However, no significant association was found between age and aggressive behavior, suggesting that age-related differences may be more evident in relative deprivation and deviant peer affiliation than in aggressive behavior.

## 5 Implications for intervention and practice

The findings of this study have important practical implications for preventing aggressive behavior among students in specialized schools.

At the school level, three complementary strategies may be considered. First, schools should foster an inclusive and supportive social environment. Positive teacher–student relationships and peer interactions can strengthen adolescents’ sense of school belonging (Allen et al., 2022). Therefore, schools may foster supportive teacher–student relationships and provide opportunities for cooperative activities and peer support to promote positive interpersonal interactions and mitigate the negative effects associated with experiences of social exclusion. Second, interventions should strengthen students’ cognitive reappraisal and emotion-regulation skills. School-based emotion-regulation interventions have been shown to improve adolescents’ emotion-regulation skills and psychological adjustment (Pedrini et al., 2022). Accordingly, cognitive reappraisal and emotion-regulation strategies may be incorporated into intervention programs to help students interpret negative interpersonal experiences more adaptively and regulate negative emotions associated with perceived unfairness, anger, and resentment, thereby potentially reducing the likelihood of aggressive behavior. Finally, schools should promote positive and supportive peer relationships. Prosocial and deviant peer affiliations may have different implications for adolescent adjustment and behavior (Yoon et al., 2025). Therefore, schools may provide more opportunities for prosocial peer interactions and incorporate social skills training and guidance on peer relationships to help students develop stable and supportive peer relationships and reduce the potential negative influence of deviant peer affiliation.

At the family level, parents or guardians should provide greater emotional support and engage in open communication, reduce rejecting responses, remain attentive to students’ experiences of relative deprivation and peer affiliations, and strengthen communication and collaboration with schools to provide continuous and stable family support.

At the societal level, communities and social service agencies should work to reduce the labeling and social exclusion of students in specialized schools. They should also provide psychological counseling, peer support, and opportunities for social participation to help these students build positive social connections. The joint involvement of schools, families, and society could contribute to a more inclusive and sustained support system, thereby reducing the risk of aggressive behavior among students in specialized schools.

## 6 Limitations and future directions

Several limitations should be considered. First, this study employed a cross-sectional design, which allows for the examination of associations and indirect relationships among variables but does not permit firm conclusions regarding causality. Future research could employ longitudinal designs to further examine the temporal and dynamic relationships among social exclusion, relative deprivation, deviant peer affiliation, and aggressive behavior. Second, the sample in this study has certain limitations. Participants were recruited from three specialized schools in Hunan, Shandong, and Hainan provinces, which may limit the generalizability of the findings. In addition, the sample was predominantly male, resulting in an imbalanced gender distribution. Future studies should expand the sampling scope to include diverse regions and different types of specialized schools, while also achieving a more balanced gender composition to enhance external validity. Finally, although demographic variables such as age and gender were controlled for in this study, aggressive behavior may also be influenced by a range of individual and contextual factors, including family socioeconomic status, family structure, and parenting styles. Future research could incorporate a broader range of individual and environmental factors to provide a more comprehensive understanding of the development of aggressive behavior.

## 7 Conclusion

This study found that social exclusion was positively associated with aggressive behavior among students in specialized schools. Relative deprivation and deviant peer affiliation independently mediated this association and also formed a serial mediating pathway. These findings deepen our understanding of the mechanisms underlying aggressive behavior among students in specialized schools and provide practical implications for prevention and intervention.

## Data Availability

The raw data supporting the conclusions of this article will be made available by the authors, without undue reservation.
